# Epitalon increases telomere length in human cell lines through telomerase upregulation or ALT activity

**DOI:** 10.1007/s10522-025-10315-x

**Published:** 2025-09-04

**Authors:** Sarah Al-dulaimi, Ross Thomas, Sheila Matta, Terry Roberts

**Affiliations:** 1 Centre for Genome Engineering and Maintenance, Division of Biosciences, Department of Life Sciences, College of Health and Life Sciences, Brunel University London, Uxbridge, UB8 3PH UK; 2 Respiratory Clinical Research Facility, Royal Brompton Hospital, Fulham Road, London, SW3 6HP UK

**Keywords:** Telomerase, ALT, Telomere length, Mammalian cells

## Abstract

Epitalon, a naturally occurring tetrapeptide, is known for its anti-aging effects on mammalian cells. This happens through the induction of telomerase enzyme activity, resulting in the extension of telomere length. A strong link exists between telomere length and aging-related diseases. Therefore, telomeres are considered to be one of the biomarkers of aging, and increasing or maintaining telomere length may contribute to healthy aging and longevity. Epitalon has been the subject of several anti-aging studies however, quantitative data on the biomolecular pathway leading to telomere length increase, *hTERT mRNA* expression, telomerase enzyme activity, and ALT activation have not been extensively studied in different cell types. In this article, the breast cancer cell lines 21NT, BT474, and normal epithelial and fibroblast cells were treated with epitalon then DNA, RNA, and proteins were extracted. qPCR and Immunofluorescence analysis demonstrated dose-dependent telomere length extension in normal cells through *hTERT* and telomerase upregulation. In cancer cells, significant telomere length extension also occurred through ALT (Alternative Lengthening of Telomeres) activation. Only a minor increase in ALT activity was observed in Normal cells, thereby showing that it was specific to cancer cells. Our data suggests that epitalon can extend telomere length in normal healthy mammalian cells through the upregulation of *hTERT* mRNA expression and telomerase enzyme activity.

**Supplementary Information:**

The online version contains supplementary material available at 10.1007/s10522-025-10315-x.

## Introduction

The average age of the world’s population is increasing year on year due to people living longer and a decline in the birth rate (Beard et al 2016). Aging itself is a multifactorial dynamic process occurring at a cellular level where cells gradually slow down and enter into what is known as replicative senescence (Di Micco et al. 2021). Aging itself leads to many health implications (Jaul et al. 2017, Tenchov et al 2024) which can reduce an individual’s quality of life and lead to complications that overburden the healthcare system (Aina et al 2021). Diseases such as Alzheimer’s, dementia, sarcopenia, arthritis, Parkinson’s, osteoporosis, maculopathy, COPD, and cancer increase with advancing age (Franceschi et al 2018). The concept of healthy aging (Menassa et al 2023; Beard et al 2016) has been developed to define and study the molecular mechanisms involved in this process (Guo et al 2022). Mechanisms such as cellular senescence, genomic instability, telomere attrition, mitochondrial dysfunction, epigenetic alterations, and loss of proteostasis all contribute to an aging phenotype (Tenchov et al 2024) and are considered hallmarks or biomarkers for aging. To try and reduce the burden of aging on society and to promote healthy aging, the pharmaceutical and supplement industries are identifying or re-purposing drugs for anti-aging intervention. Examples of pharmaceutical drugs that are considered to have anti-aging properties include Metformin, lithium, and rapamycin (Du et al 2024). They are now undergoing extensive research with human trials for potential use as part of the healthy aging process (Guarente et al 2024). The supplement and natural products industry has always had an input on the identification of compounds that have anti-aging properties (Dixit et al 2025), most notable TA-65 (Bernardes de Jesus et al 2011), resveratrol (Zhou et al 2021) and omega-3 fatty acid (Bischoff-Ferrari et al. 2025; Kiecolt-Glaser et al 2013). All 3 compounds named above affect one particular biomarker of aging, telomere attrition, they have been shown to increase or at least maintain telomere length (Zhou et al 2021; de Jesus et al 2011; Bischoff-Ferrari et al 2025). The link between telomere length, cancer, and aging has long been established (Huang et al 2025), since the discovery of the enzyme telomerase which maintains telomeres (Greider CW, Blackburn EH 1985, Feng J et al. 1995). Telomeres are found at the ends of eukaryotic chromosomes and consist of a 6-base pair DNA sequence, TTAGGG, which is tightly bound to a six-protein complex called shelterin. The function of telomeres is to protect the chromosome ends from DNA damage (Lewis and Tollefsbol 2016). The telomerase enzyme is composed of two subunits, *hTERT* and *hTR*, together, they are responsible for the synthesis of telomeric DNA. Telomerase is absent in mature somatic cells, but is reactivated in 90% of all human cancers (Shay and Bacchetti 1997). Normal, young human somatic cells have relatively long telomeres, which shorten by up to 70 bp per year, due to the end replication problem (Yik et al. 2021; Whittemore et al. 2019). Critically short telomeres prevent cell cycling and proliferation, eventually leading to cellular senescence. This phenomenon is known as the Hayflick limit (Bernardes de Jesus and Blasco 2013). Therefore, preventing telomere attrition through the expression of telomerase can increase lifespan and longevity. Telomere length is considered to be a biomarker of aging (Schellnegger et al 2024; Vaiserman and Krasnienkov 2021; Boccardi 2025) and can be used to predict organismal age. Increasing telomere length by overexpressing the enzyme telomerase may increase the lifespan of healthy mammals by up to 24% (de Jesus et al. 2012).

The tetrapeptide (AEDG) epitalon, which is also known as epithalon, was first identified in a pineal gland extract and is based on the polypeptide Epithalamin (Khavinson et al 2017; Araj et al 2025). The body naturally produces very small amounts of this peptide however, it is available as a synthetic supplement for research use only. Studies have shown that the peptide can increase the lifespan and longevity of mammalian cells (Khavinson et al 2003a, b, Khavinson et al 2000, Khavinson et al 2017, Anisimov and Khavinson 2010), through the activation of telomerase, which results in telomere length extension (Khavinson et al 2003a, b). However, there has not been a comprehensive study directly showing a quantitative increase in telomere lengths, telomerase activity, *hTERT* expression, and even ALT (Alternative lengthening of Telomeres) activity post-treatment with different doses of epitalon. This is the aim of our study.

## Materials and methods

### Cell culture and treatments

Human 21NT breast cancer cells were cultured in 1 × Modified Eagle’s medium alpha (alpha MEM) (Gibco), 2.8 μM hydrocortisone, 1 μg/ml insulin, 10% foetal calf serum (FCS), 1% gluta- max, 10 mM HEPES, 0.1 mM NEAA and 12.5 ng/ml Epithelial Growth Factor (EGF). BT474 cells and PC3-*hTERT* cells were cultured in DMEM F-12 medium (Gibco, Invitrogen) with 10% FBS (Gibco, Invitrogen) and 1% Penicillin–Streptomycin (Gibco). The PC3-*hTERT* cell line was created by stably overexpressing the full-length *hTERT* cDNA in PC3 cells (Hasan et al. 2022). This line expresses very high levels of *hTERT* mRNA and telomerase and was used to construct a standard curve for telomerase activity. U2OS cells were cultured in McCoy’s 5A with 10% FCS (Gibco, Invitrogen) and 1% glutamax, 0.5 μg/ml hydrocortisone. IBR.3 cells were cultured in RPMI 1640 medium (Biosera) with 10% FBS (Gibco, Invitrogen) and 1% Penicillin–Streptomycin (Gibco). HMEC cells were cultured in Basal medium (Mammary Life ™) containing Insulin, L-Glutamine, Epinephrine, Apo-Transferrin, rh-TGF, Extract-P, and Hydrocortisone.

Epitalon was purchased from UK Peptides. Stock solutions were prepared by dissolving 10 mg of epitalon in 4 ml of bacteriostatic water, resulting in a concentration of 2.5 mg/ml. Breast cancer cells (21NT and BT474) were treated daily with (0.1, 0.2, 0.5, and 1.0 μg/mL) of epitalon for 4 days. Normal fibroblast cells (IBR.3) and epithelial cells (HMEC) were treated daily with 1.0 μg/mL of epitalon for 3 weeks. Untreated cells were included to serve as a baseline control. Throughout both treatments, the cells were regularly monitored, and the culture media were refreshed daily.

### DNA extraction and telomere length measured by qPCR

DNA from human cell lines (21NT, BT474, IBR.3, and HMEC) was isolated using the Wizard genomic DNA purification kit and protocol from Promega (A1120). Telomere length estimation was performed using the qPCR technique (O’Callaghan and Fenech 2011). A telomeric standard curve was established by serial dilutions of the telomere standard oligomer (Table 1). A single-copy gene, 36B4, was used as a genomic DNA control and copy number determination; therefore, a serial dilution of the 34B4 standard oligomer was performed. Ct values generated from the qPCR were plotted to produce standard curves (supplementary data), which were then used to calculate the total telomere length in kb for all samples. (As described by O’Callaghan and Fenech 2011).

**Table 1:** Primer sequences

Oligomer / gene name	Oligomer sequence	Product size
36B4 standard	CAGCAAGTGGGAAGGTGTAATCCGTCTCCACAGACAAGGCCAGGACTCGTTTGTACCCGTTGATGATAGAATGGG	75
Telomere standard	(TTAGGG)14	84
Telo (F)	CGGTTTGTTTGGGTTTGGGTTTGGGTTTGGGTTTGGGTT	> 76
Telo (R)	GGCTTGCCTTACCCTTACCCTTACCCTTACCCTTACCCT	> 76
36B4 (F)	CAGCAAGTGGGAAGGTGTAATCC	75
36B4 (R)	CCCATTCTATCATCAACGGGTACAA	75
*hTERT(F)*	CGGAAGAGTGTCTGGAGCAA	200
*hTERT(R)*	GGATGAAGCGGAGTCTGGA	200
*GAPDH*(F)	GAAGGTGAAGGTCGGAGT	226
*GAPDH*(R)	GAAGATGGTGATGGGATTTC	226
Telomerase Substrate (TS)	AATCCGTCGAGCAGAGTT	
Anchored Return Primer(ACX)	GCGCGG(CTTACC)3CTAACC	

### RNA extraction, cDNA conversion, and qPCR

RNA from 21NT, BT474, IBR.3, and HMEC was extracted, and mRNA gene expression analysis was performed (primers listed in Table 1) using qPCR as described previously (Al-dulaimi et al 2024). Analysis of the RT-qPCR data was performed using the delta delta Ct method, and graphs show the relative quantity of gene expression (RQ).

### Telomerase activity measured by telomere repeat amplification protocol (TRAP)

The TRAP assay was used to determine telomerase activity. Protein from the 21NT, BT474, HMEC and IBR.3 cells were extracted using the TRAPeze 1 × CHAPS lysis buffer (S7705, Millipore) and quantified using the CB-X protein assay kit (G-Bioscience). For estimation of the telomerase activity, the procedure outlined in (Al-dulaimi et al 2024) was followed. A serial dilution of proteins from 500 to 50 ng from the PC3-*hTERT* cell line (telomerase-positive prostate cancer cell) was used to construct a standard curve of telomerase activity (supplementary data). A negative control was also included for enzyme activity by heating 200 ng of the PC3 protein to 95 ℃/10 min to inactivate the telomerase enzyme. For each qPCR reaction, 25 μl of master mix was prepared by adding 12.5 μl of the 2 × Universal SYBR (ThermoFisher) 5.5 μl of RNAse-free water, 1 μl ACX primer (0.05 μg /ul), 1 μl TS primer (0.1 μg /μl) (Table 1), and 4 μl of the protein sample. The reactions were incubated at 25^◦^C for 20 min to allow telomerase to synthesise the TRAP ladders, then the qPCR was carried out at 95^◦^C for 10 min and 35 cycles of 95 ℃ for 30 s, and 60 ℃ for 90 s. Telomerase activity was quantified using the PC-3 *hTERT* standard curve and QuantStudio V1.3 software.

### ALT activity quantified using the C-circle assay

C-circle assay was performed as previously described (Al-dulaimi et al 2024). 30 ng of genomic DNA was diluted in 10 mM TRIS (pH 7.6) buffer. The diluted genomic DNA was added to a 10 μl reaction mix containing 0.2 mg/ml BSA, 4 mM DTT, 0.10% Tween, 0.1 mM dTTP, 1X phi29 buffer, and 15 U phi 29 DNA polymerase. Reactions without the phi 29 polymerase enzyme were included as a negative control. Samples were incubated for 8 h at 30 ℃ followed by 65 ℃ for 20 min. The levels of telomeric DNA in samples were quantified using qPCR, the reference gene used was the single-copy gene 36B4, with primers for both telomeric and 36B4 sequences outlined in Table 1. The qPCR cycling conditions were as follows: an initial denaturation at 95 °C for 15 min, followed by 30 cycles of 95 °C for 7 s and 58 °C for 10 s, and a final extension step at 95 °C for 5 min, then 40 cycles of 95 °C for 15 s and 58 °C for 30 s. The C-circle assay was performed using qPCR QuantStudio V1.3 software. Standard curves for the telomere standard and reference gene were used to calculate telomere lengths in kilobases.

### Immunofluorescence (IF) for PML bodies

21NT cells (treated with 1ug/ul epitalon and untreated) were plated onto glass microscope slides (Thermo Scientific), then fixed in 2% formaldehyde (Fisher Scientific) for 15 min and cold methanol for 10 min. Following this, they were washed with PBS and permeabilized with 0.3% (v/v) Triton X-100 (Sigma-Aldrich) solution for 5 min. Cells were washed with PBS and blocked with 5% BSA for 1 h at RT. Cells were then incubated with a mouse monoclonal primary antibody against PML (PG-M3): sc-966) diluted in blocking solution for 2 h at RT. Following this, they were washed three times with PBST (1X PBS and 0.05% Tween-20), then incubated with mouse IgG FITC conjugated secondary antibody (Invitrogen). After a one-hour incubation, the cells were washed three times with PBST and stained with DAPI. For each slide, 100 cells were counted using Lecia microscope with a 100 X objective to confirm the presence of PML bodies.

### Statistical analysis

All statistical analyses were carried out using GraphPad Prism (GraphPad Software). Statistical analysis for multiple samples was performed using the one-way ANOVA p < 0.05. Also, unpaired student’s test *P < 0.05, **P < 0.01, ***P < 0.001, ****P < 0.0001 was used for 2-way analysis.

## Results

### Epitalon increases telomere length

A dose–response experiment was carried out to detect the impact of various concentrations of epitalon on telomere length in the 21NT and BT474 breast cancer cells. Both cell lines were treated with concentrations ranging from 0.1, 0.2, 0.5 to 1 μg/ml for 4 days. DNA was extracted and telomere length was measured using qPCR as described above.

**Fig. 1:**
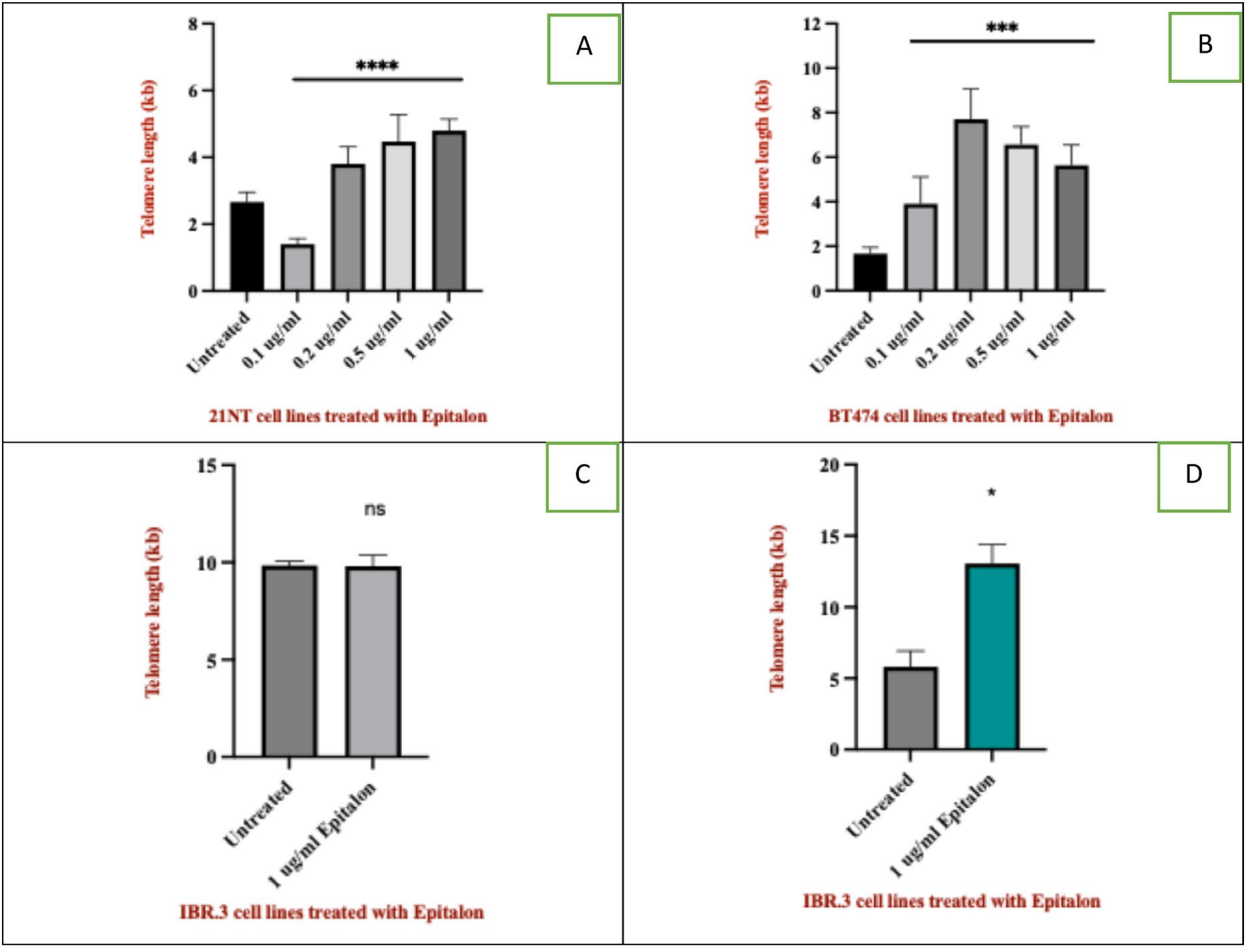
Telomere length of breast cancer cells and normal cells treated with epitalon. **A** 21NT cells **B** BT474 cells. Both cells were treated for 4 days with varying concentrations of epitalon. (0.1, 0.2, 0.5 and 1 ug/ml). Untreated cells were used as a control. **C** Telomere length of IBR.3 cells (P.14) treated with 1 μg /ml epitalon for 3 weeks. **D** telomere length of HMEC (P.14) treated with 1 μg /ml epitalon for 3 weeks. Untreated cells were included as a control

As shown in Fig. 1A and B, the treatment with epitalon for 4 days was associated with a dose-dependent increase in telomere length starting from the lower concentration of 0.2 to the highest concentration of 1 μg/ml in 21NT and BT474. The treatment depicted significant telomere extension from 2.4 kb to 4 kb with doses of 0.5 and 1 μg /ml in 21NT. In BT474, telomere length reached a maximum of 8 kb at a dose of 0.2 μg /ml. It was interesting to note that, at the higher concentrations of 0.5ug/ml and 1 μg/ml, we observed lower rate of telomere extension for BT474 when compared to the maximum length obtained at 0.2ug/ml. This did not occur in 21NT. However, at the lowest concentration of epitalon (0.1ug/ml), we observed a decrease in telomere length for 21NT, and a lower rate of telomere elongation for BT474. It suggests that low concentrations of epitalon could have an inhibitory effect on cancer cells and telomere length extension.

To test epitalon on primary cells, IBR.3 was treated with 1 μg/ml for four days. No increase in telomere lengths was observed between the untreated and treated groups (data not shown). When we extended the treatment period to three weeks, as shown in Fig. 1C for IBR.3 and Fig. 1D for HMEC, there was a significant increase in telomere length for both in comparison with the untreated control cells. It is important to note that the qPCR method used measures average telomere lengths across all chromosomes, which represents a limitation of the actual technique. It would be interesting to know if epitalon increases telomere length on all chromosomes at the same or similar rate.

### Epitalon upregulates *hTERT* expression in all cell types

Telomerase expression and ALT activity are the two mechanisms responsible for increasing telomere lengths in mammalian cells. In addition, previous publications observed an increase in telomerase activity post treatment with epitalon (Khavinson et al 2003a, b). The *hTERT* mRNA codes for the catalytic subunit of telomerase; therefore, we quantified the expression level of this gene after epitalon treatment.

**Fig. 2:**
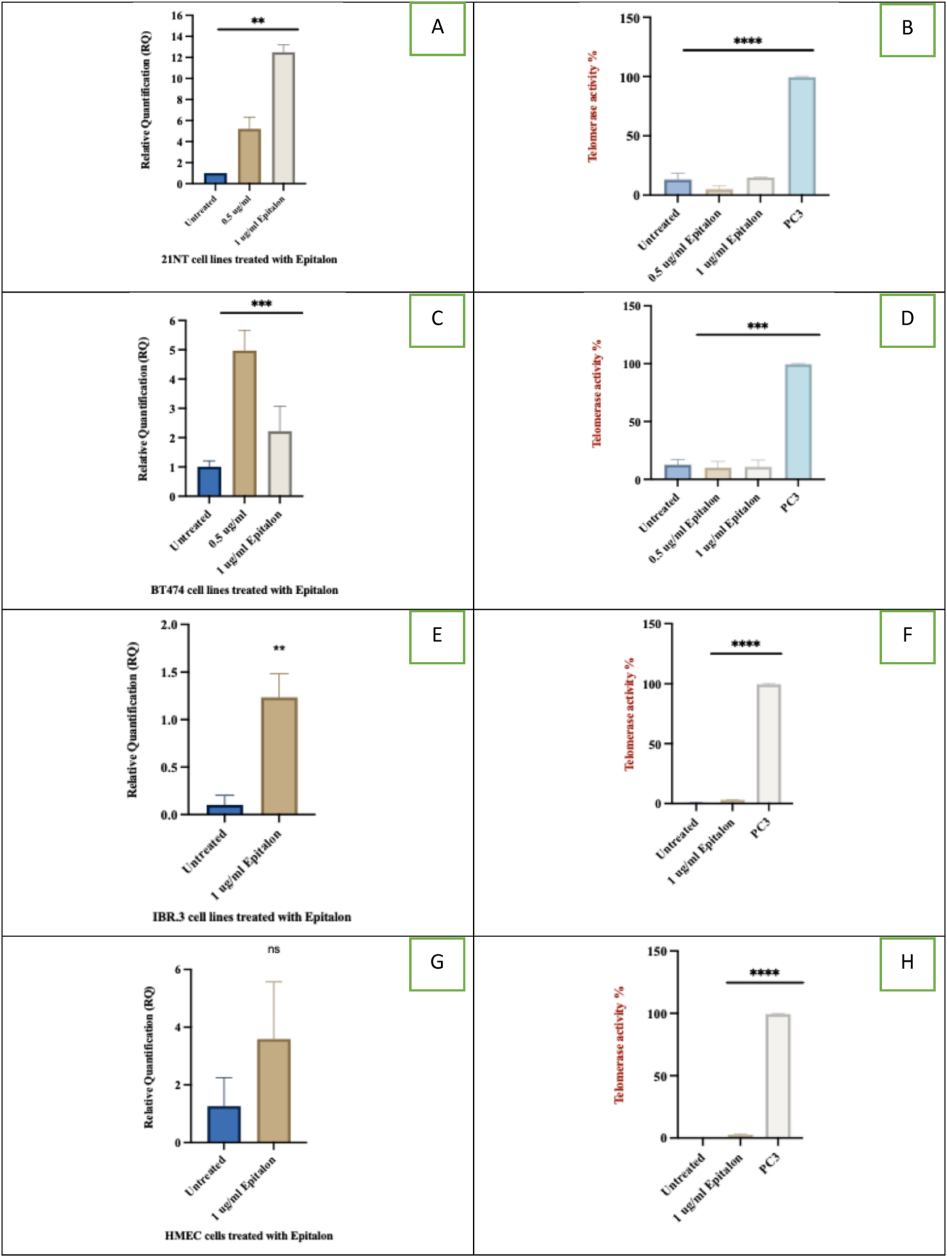
hTERT expression (RQ) and telomerase enzyme activity in breast cancer cells and normal fibroblast cells treated with epitalon. **A** and **B** hTERT expression and telomerase activity for 21NT treated with 0.5 and 1 μg/ml of epitalon for 4 days. **C** and **D** hTERT expression and telomerase activity of BT474 treated with 0.5 and 1 μg/ml of epitalon for 4 days. PC3 was included as a positive control for telomerase activity. **E** and **F** *hTERT* expression and telomerase activity for IBR.3 were treated with 1 μg/ml of epitalon for three weeks. **G** and **H** *hTERT* expression and telomerase activity for HMEC treated with 1 ug/ml of epitalon for three weeks

21NT and BT474 were treated with 0.5 and 1 μg /ml of epitalon for 4 days, RNA extracted and *hTERT* mRNA expression levels were quantified using qPCR. At 1 μg/ml, *hTERT* expression was upregulated 12-fold in 21NT and at 0.5 μg/ml, BT474 showed a fivefold upregulation relative to the untreated cells (Fig. 2A and C, respectively). IBR.3 (Fig E) and HMEC (Fig. 2G) both elevated the expression of *hTERT* mRNA after a 3-week incubation with 1ug/ml epitalon. The increase in *hTERT* seen was lower than what was obtained in the cancer cell lines, suggesting that normal cells have a more robust pathway for telomerase regulation which needs to be overcome for full expression of *hTERT*.

### Epitalon elevates telomerase activity in normal cells but not cancer cells

We have shown that epitalon treatment resulted in a significant increase in telomere length for all cell lines tested (Fig. 1), which correlated with an increase in *hTERT* expression (Fig. 2A, C, E, G. As *hTERT* codes for the catalytic subunit of the functional telomerase enzyme, telomerase activity was quantified using qPCR to determine if epitalon enhances its activity through *hTERT* expression. A positive telomerase control (PC3) was included in the qPCR assay, and the negative control was a heated sample of PC3-*hTERT* this pre-heating inactivates the telomerase enzyme.

Treatment of 21NT and BT474 breast cancer cells with 0.5 and 1 μg/ml of epitalon for 4 days did not lead to a significant increase in telomerase activity compared to the non-treated controls and the telomerase positive PC3 (Fig. 2, B and D). Results indicated that although telomere length and *hTERT* were elevated after treatment with epitalon, actual telomerase activity did not follow the same pattern.

In contrast, IBR.3 and HMEC, exhibited a significant increase in telomerase activity (Fig. 2 F and H) after the 3-week incubation. Although telomerase activity was elevated in the normal cells HMEC and IBR.3 after epitalon treatment, the levels were not as high as what is seen in the untreated or treated cancer cells. Our results suggest that epitalon has a positive effect on *hTERT* expression and telomerase activity in normal cells (IBR3 and HMEC), whereas in cancer cells (BT474 and 21NT), *hTERT* is elevated however, telomerase activity is not significantly enhanced.

### Epitalon significantly increases ALT activity in cancer cells but not in normal cells

**Fig. 3:**
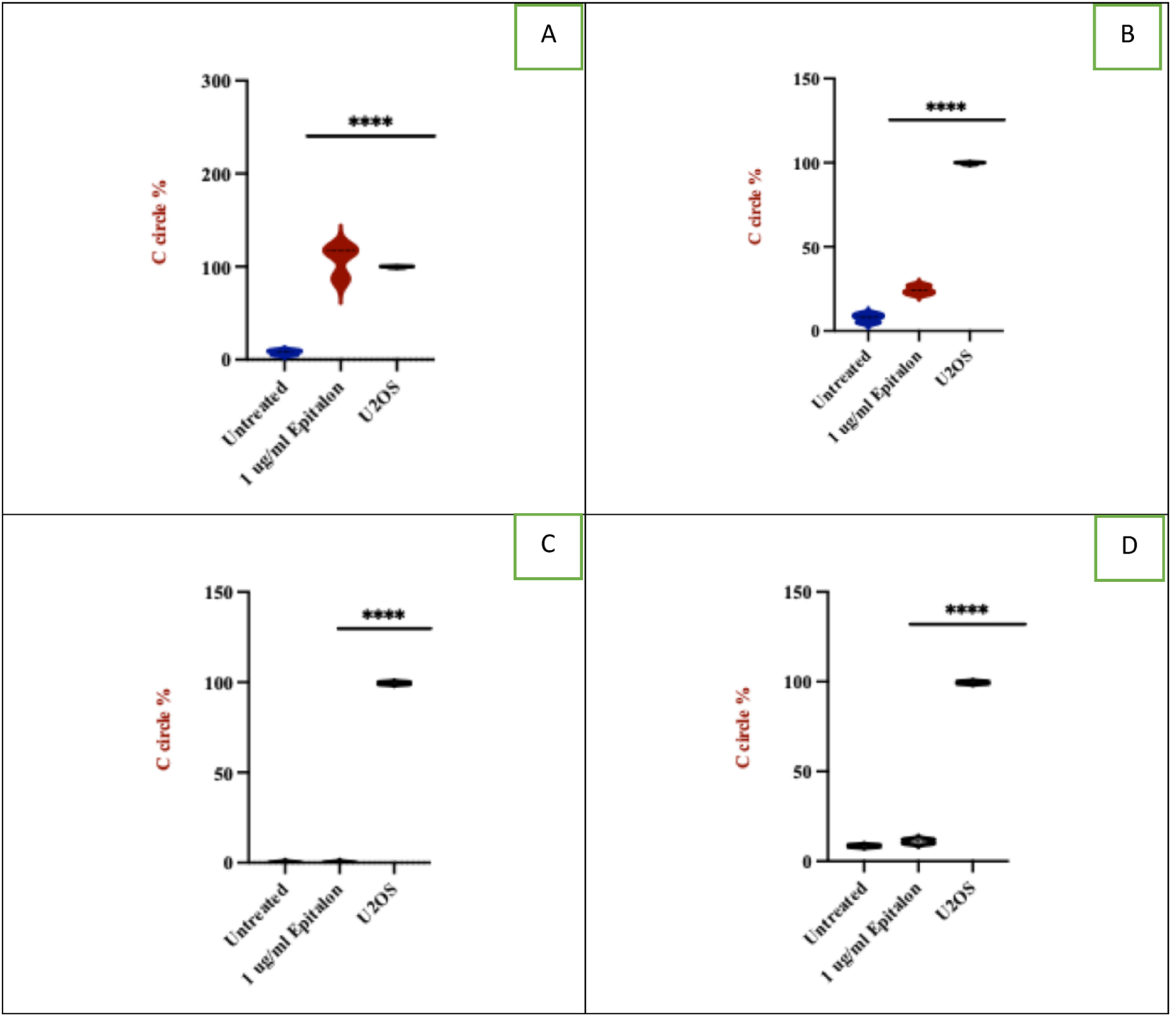

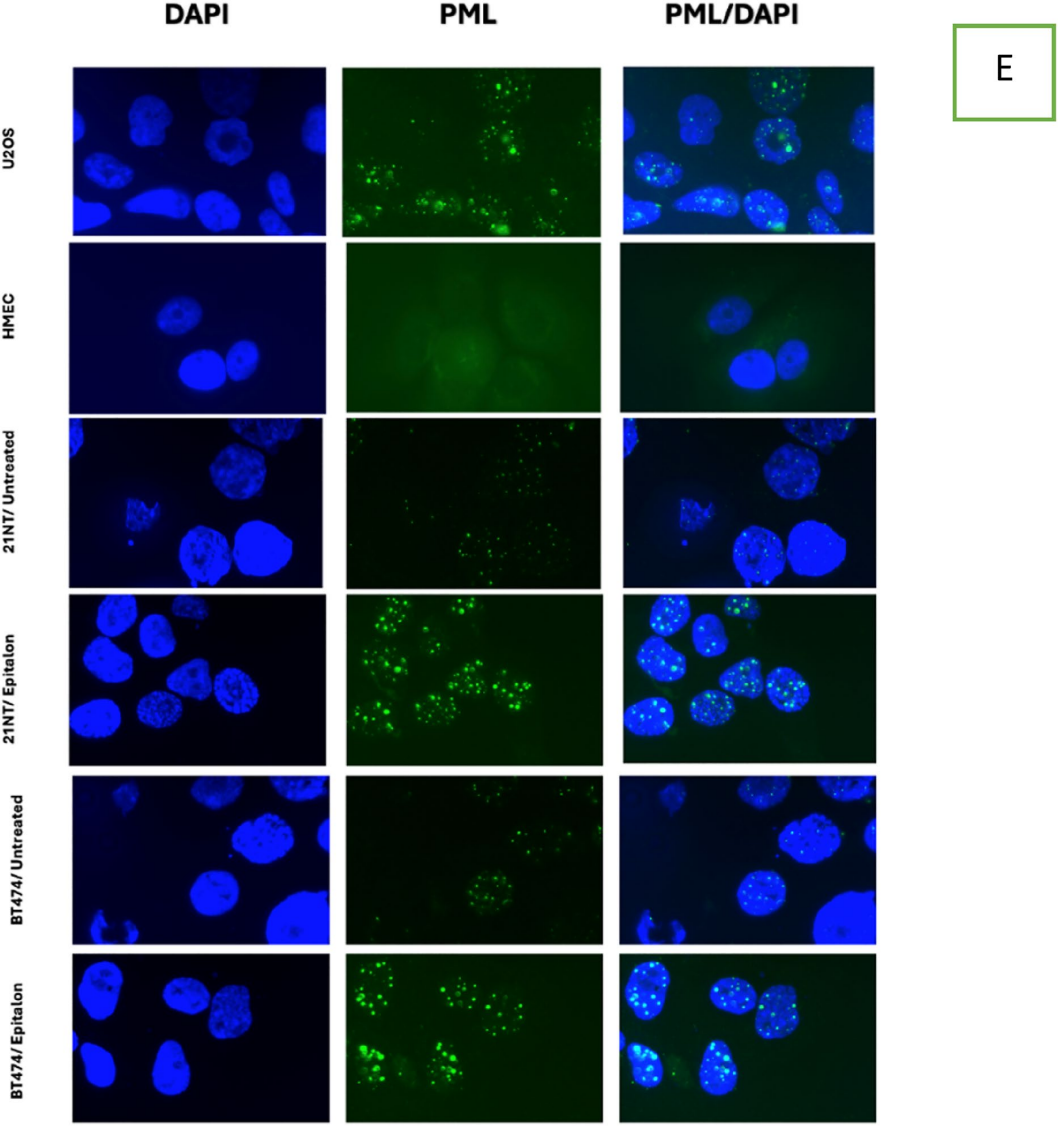
ALT activity in breast cancer cells and normal cells treated with epitalon. **A** and **B** ALT activity in 21NT and BT474 treated with 1 μg/ml of epitalon for 4 days. Untreated cells and the ALT positive U2OS were included as controls. **C** IBR.3 and **D** HMEC treated with 1 μg/ml of epitalon for three weeks. **E** Immunofluorescence to detect PML bodies in 21NT and BT474 cells treated with epitalon for 4 days. The colocalization PML bodies (green staining) within the nucleus (blue staining) for 21NT and BT474 treated with epitalon indicated the presence of ALT. PML bodies were detected using Lecia microscope with X 100 objective

We have shown that epitalon treatment did not significantly increase telomerase activity in breast cancer cells but enhanced the activity in normal cells (Fig. 2). We hypothesised that the telomere length elongation seen in the cancer cell lines may be due to ALT activity. Therefore, we quantified ALT activity using the c-circle assay and confirmed the result with immunofluorescence (IF) to detect PML bodies. 21NT and BT474 were treated with 1 μg/ml epitalon for 4 days. The ALT positive U2OS cells were included as a positive reference control, and C-circles were presented as percentages relative to the U2OS cell line (Henson et al. 2017). As shown in Fig. 3A, a substantial ten-fold increase in ALT activity was observed for 21NT after treatment with epitalon when compared with the untreated control. A lower, but still significant three-fold increase in ALT also occurred in BT474. In contrast to the cancer cells, IBR.3 showed no increase in ALT activity after treatment and HMEC exhibited an insignificant increase after treatment (Fig. 3C, D). To confirm the C-circle results, IF was used to identify and quantify PML bodies. The presence of PML bodies characterises ALT activity in cells (Chung et al 2012). IF revealed that the treatment with epitalon was associated with a significant elevation of PML bodies for both BT474 and 21NT compared to the untreated controls (Fig. 3E).

## Discussion

Telomere length is considered to be a marker of biological aging (Vaiserman* and Krasnienkov 2021), and telomere length maintenance can increase lifespan and longevity in mammals (Bernardes de Jesus et al 2012). Epitalon was initially shown to induce telomerase activity and elongate telomeres in human somatic cells by Khavinson in 2003. It was shown to stimulate the proliferative capacity of epithelial cells (Khavinson et al 2003a, b) and fibroblast (Lin’kova et al 2016) in vitro thereby increasing the lifespan of the cells. However, very little quantitative data has ever been published on comparing telomere lengths, *hTERT* expression, telomerase and ALT activity in normal and cancer cells treated with this tetrapeptide.

Our results indicate that epitalon increases telomere lengths of both cancer (21NT and BT474, Fig. 1) and normal cells (IBR.3 and HMEC, Fig. 1) *invitro*. However, normal cells need a longer, 3-week incubation compared to the 4 days that cancer cells require. Normal cells may require a longer incubation period for telomere elongation as they do not possess active telomere maintenance mechanisms. Epitalon will have to activate these mechanisms and several rounds of cellular division may be required before we see any increase in telomere length. Both cancer cell lines used are telomerase positive, and have telomere maintenance mechanisms already active. Therefore, increasing telomere lengths may occur more readily.

*hTERT* expression and telomerase activity were then quantified in cells treated with the tetrapeptide. All cell types upregulated *hTERT*, with the highest increases being seen in the cancer cell lines (Fig. 2). This is to be expected as these cells are already expressing *hTERT* mRNA, moreover, in the normal cells, epitalon would have to initiate *hTERT* expression, presumably by promoter activation. Cancer cell lines showed no significant increase in telomerase enzyme activity (Fig. 2) which was a surprise as *hTERT* expression is strongly linked to telomerase activity (Leao et al. 2018). It’s known that 22 splice variants of *hTERT* exist (Plyasova and Zhdanov 2021) however, only the full-length variant codes for the fully active telomerase enzyme and some variants can act in a dominant negative way to inhibit telomerase activity. Therefore, even though we obtained significant increases in the *hTERT* mRNA expression for 21NT and BT474 after epitalon treatment, lower levels of the fully functional variant may have been produced hence no correlation between mRNA levels and telomerase enzyme activity was observed. In addition, studies have shown that only pre-spliced mRNA containing intron 2 codes for fully functional telomerase (Ducreast et al. 2001). In normal cells, however, epitalon led to a significant increase in telomerase activity by fourfold in IBR.3 treated cells compared to untreated, and 26-fold increase in treated HMEC cells compared to untreated. (Fig. 2). When comparing telomerase enzyme activity in the cell lines used (normal and cancer), it’s clear to see that the increase in telomerase activity seen in the normal cells treated with epitalon, does not reach the levels seen in the cancer cell lines (Fig. 2). This is a significant finding as it suggests that the normal cells treated with epitalon will not gain full immortality and an extended lifespan as seen in cancer cells. Full transformation of normal cells to a cancerous phenotype is a multi-step process (Hanahan and Weinberg 2011; Fouad and Aanei 2017, Hanahan et al. 2022) which requires the deactivation of tumour suppressor genes such as P53, RB and p16 (Dimri et al 2005) and/or activation of oncogenes. Telomerase then acts to stabilise the telomeres and prevent them from shortening thereby allowing the cells to gain immortality.

Following the finding that the cancer cell lines did not elevate telomerase enzyme activity upon epitalon treatment but did elongate telomeres, we investigated the other commonly used mechanism to extend telomeres in mammals, ALT (Alternative Lengthening of Telomeres). To our surprise, the c-circle results revealed that both cancer cell lines dramatically increased ALT activity to a level higher than what was seen in the untreated controls (Fig. 3). In contrast, the normal cells (IBR.3 and HMEC) showed little (HMEC) or no (IBR.3) ALT activation (Fig. 3). To confirm the results for the cancer cell lines, we performed IF to detect PML bodies, a marker of ALT. Again, both 21NT and BT474 showed an increase in PML post treatment with epitalon to levels higher than the untreated controls (Fig. 3), suggesting that ALT has been activated and is responsible for the increase in telomere length seen in the cancer cells. It is interesting to note that untreated telomerase positive cells contained PML bodies, this confirms that the ALT mechanism was present before exposure to epitalon. Both mechanisms of telomere length maintenance, telomerase and ALT activity, are known to co-exist in cancer cells (De Vitis et al 2018; Hu et al 2016; Perrem et al 2001), and our data suggest that epitalon activates ALT in cancer cells only and not in normal cells. When comparing the C-Circle and PML results for the ALT positive U2OS cells, it was clear to see that the the levels did not match. The C-circle levels were much higher that what was shown by the PML bodies. Using a PCR based mythology can lead to overestimation of the results due to the sensitivity of the technique.

Epitalon has been shown to bind preferentially to methylated cytosine in DNA (Fedoreyeva et al. 2011, Khavinson et al 2008), and with the linker histone protein H1 (H1.3 and H1.6) thereby influencing epigenetic regulation and expression of genes (Khavinson et al 2020). Given the interaction between epitalon, DNA and histone proteins, we speculate that the tetrapeptide may trap proteins on the DNA and induce ALT activity. Rose et al 2023 demonstrated that certain chemotherapeutic drugs such as talazoparib, camptothecin and etoposide binds and traps proteins (PARP and topoisomerases I and II, respectively) on DNA strands, resulting in DNA damage and double-strand breaks. In cells lacking ATRX, a gene involved in facilitating double strand break repair and preventing replication fork stalling (Huh et al 2016, Raghunandan et al. 2020), Break-Induced repair (O Mori et al 2024) can initiate ALT activity. While we have no data showing this, the binding activity of epitalon may induce similar effects, thereby initiating ALT in cancer cells.

In addition to this, the binding of epitalon to histone H1.3/1.6 may lead to epigenetic regulation or inhibition of the histone’s function (similar to protein trapping) in cells thereby leading to the induction of ALT. Recent studies looking at the telomeric proteome during replication observed high levels of histone protein H1 at telomeres during replication (Lin et al 2021), and cells that had H1 depleted through knockout experiments are sensitive to DNA damage and double strand breaks (Murga et al 2007). These cells show an increase in telomeric sister chromatid exchange (T-SCE) and rapid telomere elongation using ALT recombination mechanisms (Murga et al 2007). This suggests that Histone H1 is vital for telomere stability and the binding of epitalon to H1.3 and H1.6 could inhibit its function thereby triggering ALT. The activation of ALT can lead to suppression of telomerase activity (O’Sullivan et al 2014) hence, we observed no increase in telomerase enzyme activity in the cancer cells after treatment with epitalon. To account for the induction of ALT in the breast cancer cells (21NT and BT474) and non-induction in normal cells (IBR3 and HMEC), it is known that histone H1 is expressed at higher physiological levels in normal tissues and lower levels in breast cancer cells (Scaffidi 2016). Depletion of H1 promotes oncogenic and self-renewing activity within cells due to decompaction of chromatin and gene activation (Torres et al 2016). Indeed, ALT telomeres have a relaxed telomeric chromatin configuration (compared to telomerase positive and normal cells) making them more susceptible to DNA damage and telomere elongation through ALT (Lin et al 2021; O’Sullivan et al 2014). Therefore, cancer cells may be more susceptible to ALT activation due to the binding of epitalon to the already reduced levels of histone H1, whereas normal cells may be more resilient as they naturally express higher levels of H1.

Histone H1 is involved in the regulation of telomerase activity in cancerous cells. Overexpression of Histone H1.3 was shown to suppress the growth of ovarian cancer cells, H1.3 also suppresses the expression of the noncoding gene *H19* (Medrzycki, et al 2014). *H19* encodes two conserved miRNAs within its first exon and has been shown to be a telomerase regulator (El Hajj et al 2018). *H19* expression downregulates telomerase enzyme activity by binding to and disrupting the hTERT-*hTR* interaction. Disruption of the telomerase complex reduces telomerase activity but does not affect *hTERT* mRNA expression directly. Therefore, if epitalon binds to and inhibits H1.3 in the cancer cells, *H19* would be derepressed. The higher expression of *H19* will then inhibit telomerase activity in cancer cells (without affecting *hTERT* expression), thereby allowing ALT to continue telomere maintenance. As epitalon has been shown to bind to H1 (Khavinson et al 2020) it is possible that peptide may downregulate telomerase in cancer cells (through H1 binding and H19 upregulation), and activate ALT (through protein trapping) at the same time.

Telomerase activity has been extensively explored as the telomere length maintenance mechanism that can be manipulated to increase longevity. Pharmaceutical intervention has focused on upregulating the expression of telomerase to increase life span and help with healthy aging (Du et al 2024, Dixit et al 2025, Bernardes de Jesus et al. 2012, Zhou et al., Ferrari et al. 2025, Kiecolt-Glaser et al 2013). Recently, it was demonstrated that ALT was the only mechanism available for telomere lengthening and maintenance in *Alligator sinensis* and the newt *Pleurodeles waltl* (Guo et al 2023a, b; Yu et al 2022). Both vertebrates lacked telomerase activity and both have a high regenerative potential and are long-lived; they show no significant telomere erosion. When studying anti-aging drugs and supplements which focus on telomere length maintenance, we should consider both mechanisms, telomerase and ALT.

## Conclusion

This study confirms the previous results that epitalon increases telomere lengths in normal epithelial and fibroblast cells through the up-regulation of telomerase. We have provided quantitative data showing this for 2 normal human cell lines. Unexpectedly, we also observed telomere length increase in two telomerase-positive cancer cell lines however, this was found to occur through ALT activation. Importantly, ALT was not activated in normal cells. This would suggest that epitalon can be safely used in healthy individuals to maintain telomeres and thereby influence the aging process. There were several limitations of our work, the main one being that this was an in vitro study using human cell lines in 2D cell cultures. Future work should focus on 3D cultures and in vivo animal models that more accurately mimic a natural cellular environment, allowing a true evaluation of the peptides’ effectiveness as an anti-aging compound.

## Supplementary Information

Below is the link to the electronic supplementary material.

Supplementary file1 (PDF 387 KB)

## Data Availability

No datasets were generated or analysed during the current study.
