# Correction: Epitalon increases telomere length in human cell lines through telomerase upregulation or ALT activity

**DOI:** 10.1007/s10522-025-10326-8

**Published:** 2025-11-15

**Authors:** Sarah Al-dulaimi, Ross Thomas, Sheila Matta, Terry Roberts

**Affiliations:** 1 Centre for Genome Engineering and Maintenance, Division of Biosciences, Department of Life Sciences, College of Health and Life Sciences, Brunel University London, Uxbridge, UB8 3PH UK; 2 Respiratory Clinical Research Facility, Royal Brompton Hospital, Fulham Road, London, SW3 6HP UK

**Correction to**: Biogerontology 10.1007/s10522-025-10315-x

In this article, the wrong figures appeared in Figs. 1, 2 and 3. The incorrect and correct versions of Figs. 1, 2 and 3 are provided below:

Incorrect version of Fig. 1:

**Figure:**
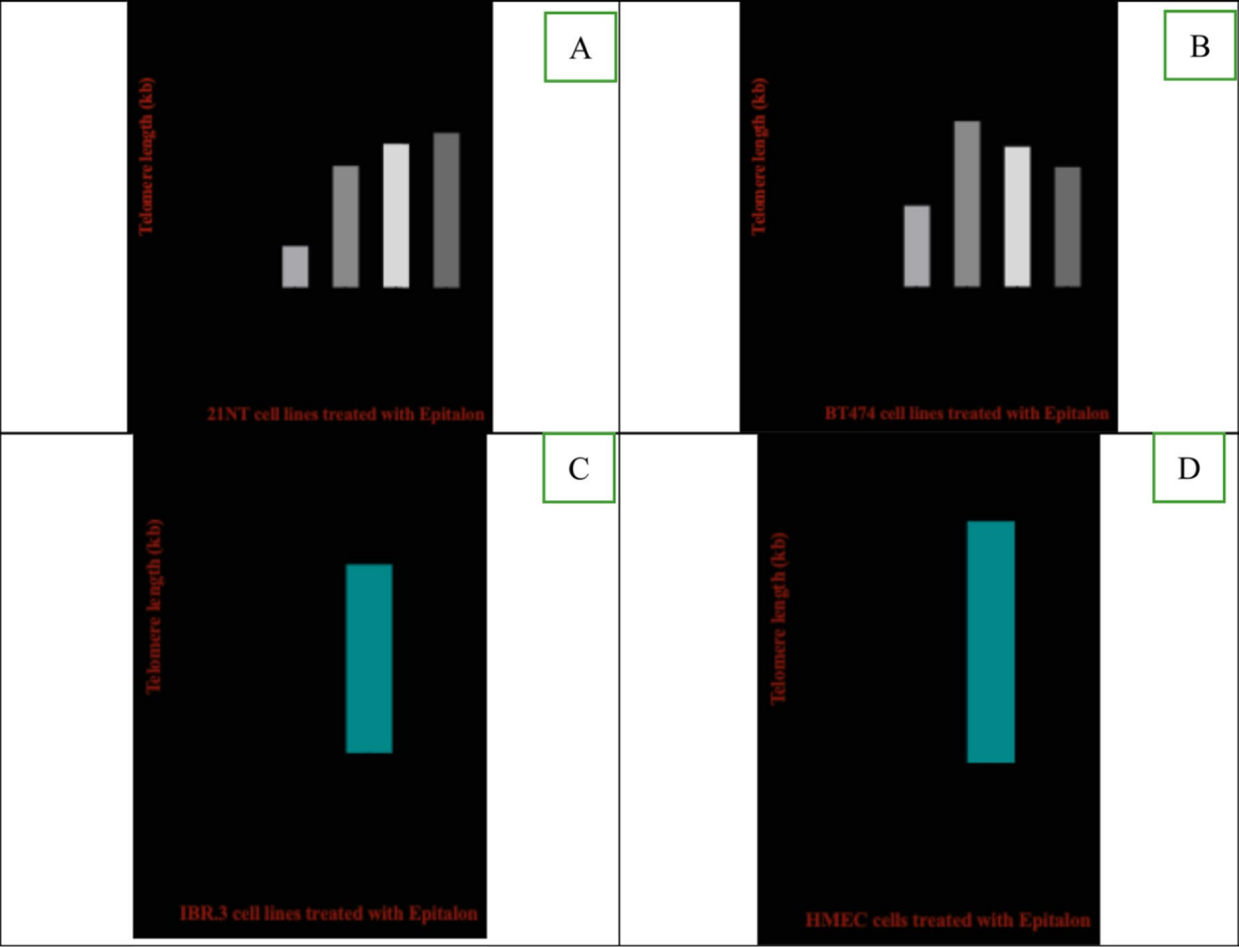
Telomere length of breast cancer cells and normal cells treated with epitalon. **A** 21NT cells **B** BT474 cells. Both cells were treated for 4 days with varying concentrations of epitalon. (0.1, 0.2, 0.5 and 1 ug/ml). Untreated cells were used as a control. **C** Telomere length of IBR.3 cells (P.14) treated with 1 μg /ml epitalon for 3 weeks. **D** telomere length of HMEC (P.14) treated with 1 μg /ml epitalon for 3 weeks. Untreated cells were included as a control

Correct Version of Fig. 1:

**Fig. 1:**
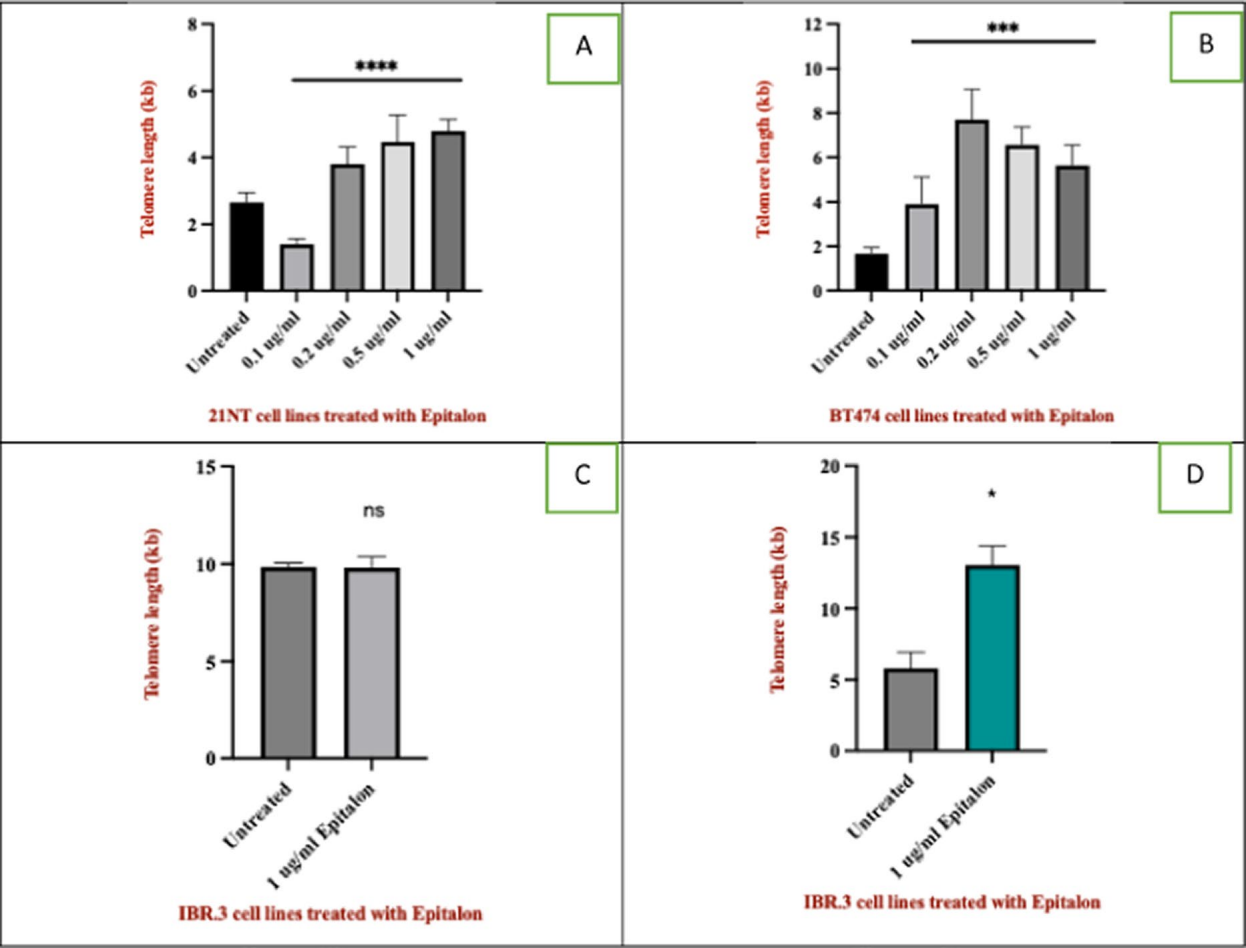
Telomere length of breast cancer cells and normal cells treated with epitalon. **A** 21NT cells **B** BT474 cells. Both cells were treated for 4 days with varying concentrations of epitalon. (0.1, 0.2, 0.5 and 1 ug/ml). Untreated cells were used as a control. **C** Telomere length of IBR.3 cells (P.14) treated with 1 μg /ml epitalon for 3 weeks. **D** telomere length of HMEC (P.14) treated with 1 μg /ml epitalon for 3 weeks. Untreated cells were included as a control

Incorrect Version of Fig. 2:

**Figure:**
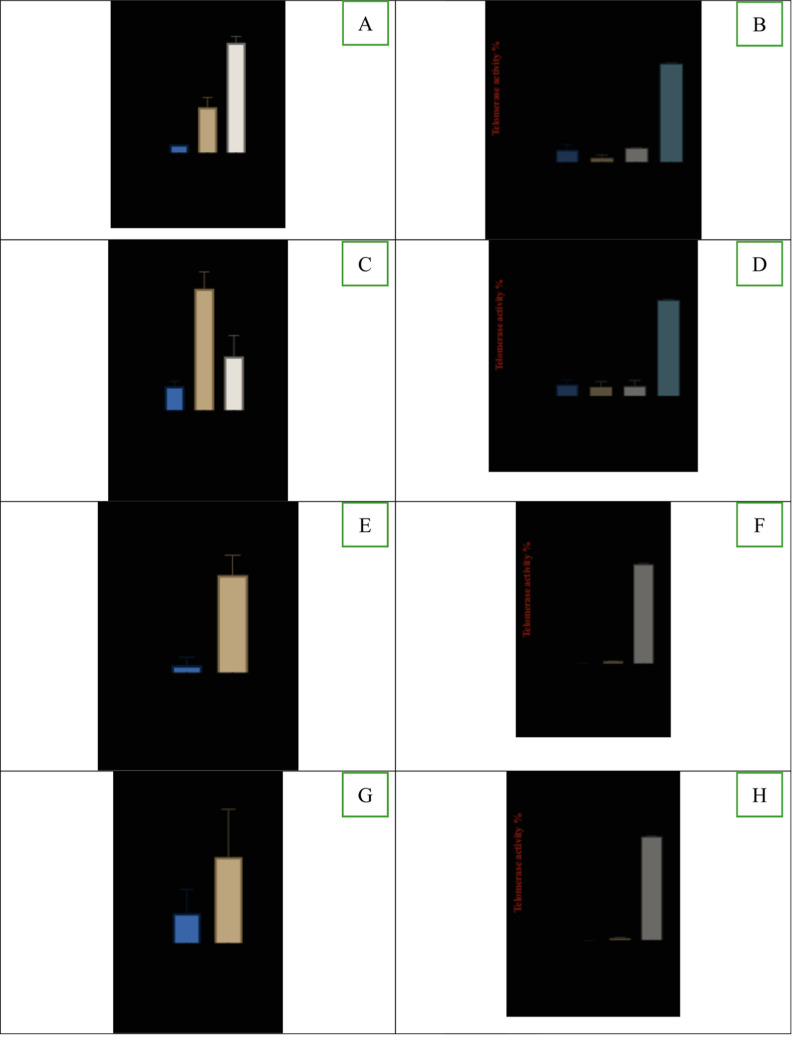
hTERT expression (RQ) and telomerase enzyme activity in breast cancer cells and normal fibroblast cells treated with epitalon. **A** and **B** hTERT expression and telomerase activity for 21NT treated with 0.5 and 1 μg/ml of epitalon for 4 days. **C** and **D** hTERT expression and telomerase activity of BT474 treated with 0.5 and 1 μg/ml of epitalon for 4 days. PC3 was included as a positive control for telomerase activity. **E** and **F** *hTERT* expression and telomerase activity for IBR.3 were treated with 1 μg/ml of epitalon for three weeks. **G** and **H** *hTERT* expression and telomerase activity for HMEC treated with 1 ug/ml of epitalon for three weeks

Correct Version of Fig. 2:

**Fig. 2:**
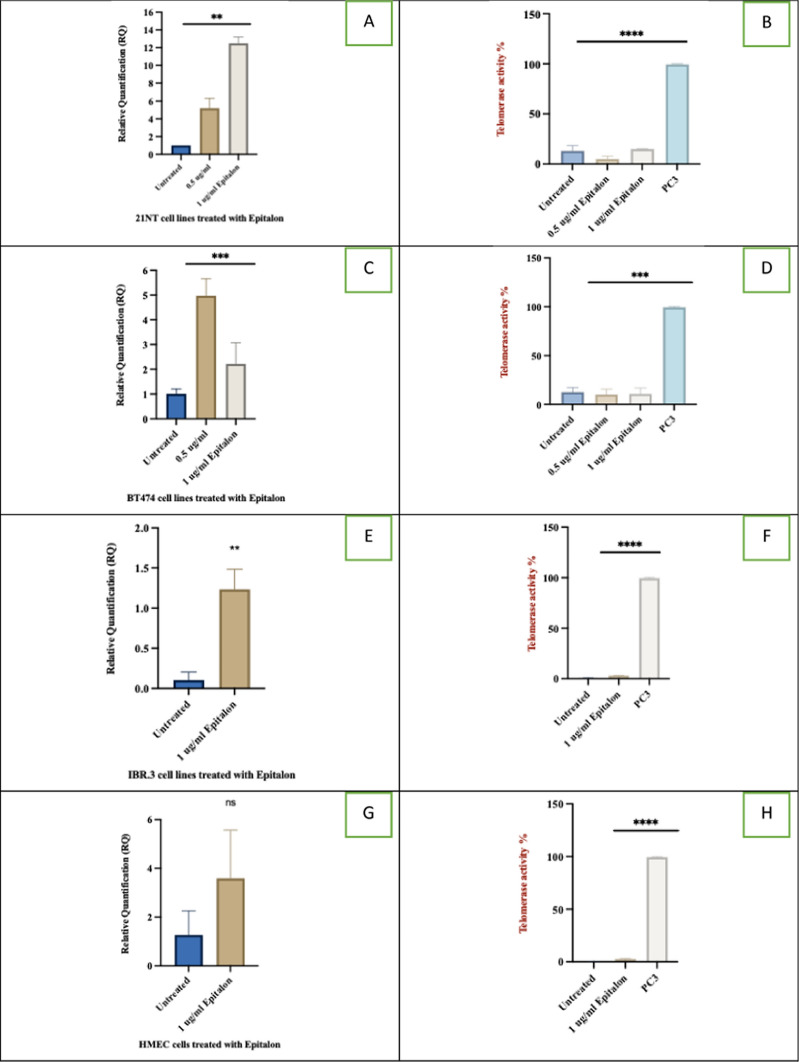
hTERT expression (RQ) and telomerase enzyme activity in breast cancer cells and normal fibroblast cells treated with epitalon. **A** and **B** hTERT expression and telomerase activity for 21NT treated with 0.5 and 1 μg/ml of epitalon for 4 days. **C** and **D** hTERT expression and telomerase activity of BT474 treated with 0.5 and 1 μg/ml of epitalon for 4 days. PC3 was included as a positive control for telomerase activity. **E** and **F** *hTERT* expression and telomerase activity for IBR.3 were treated with 1 μg/ml of epitalon for three weeks. **G** and **H** *hTERT* expression and telomerase activity for HMEC treated with 1 ug/ml of epitalon for three weeks

Incorrect Version of Fig. 3:

**Figure:**
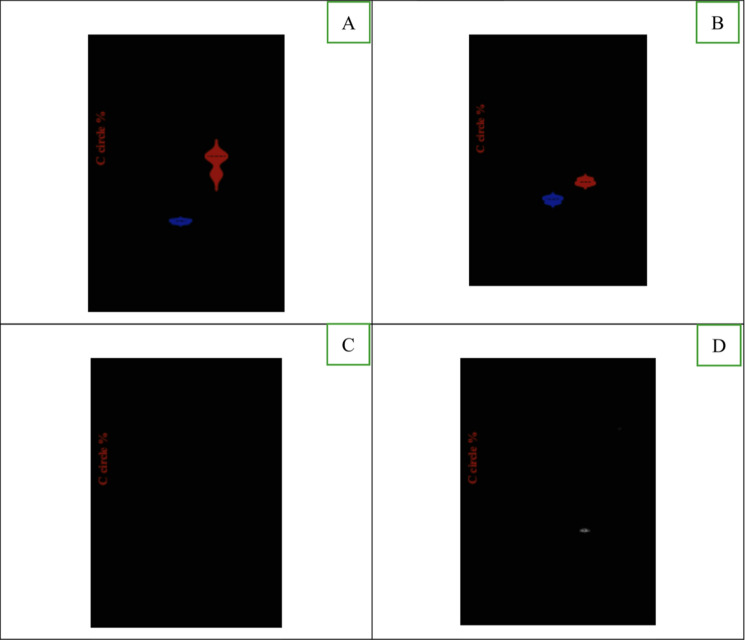
ALT activity in breast cancer cells and normal cells treated with epitalon. **A** and **B** ALT activity in 21NT and BT474 treated with 1 μg/ml of epitalon for 4 days. Untreated cells and the ALT positive U2OS were included as controls. **C** IBR.3 and **D** HMEC treated with 1 μg/ml of epitalon for three weeks. **E** Immunofluorescence to detect PML bodies in 21NT and BT474 cells treated with epitalon for 4 days. The colocalization PML bodies (green staining) within the nucleus (blue staining) for 21NT and BT474 treated with epitalon indicated the presence of ALT. PML bodies were detected using Lecia microscope with X 100 objective

Correct Version of Fig. 3:

**Fig. 3:**
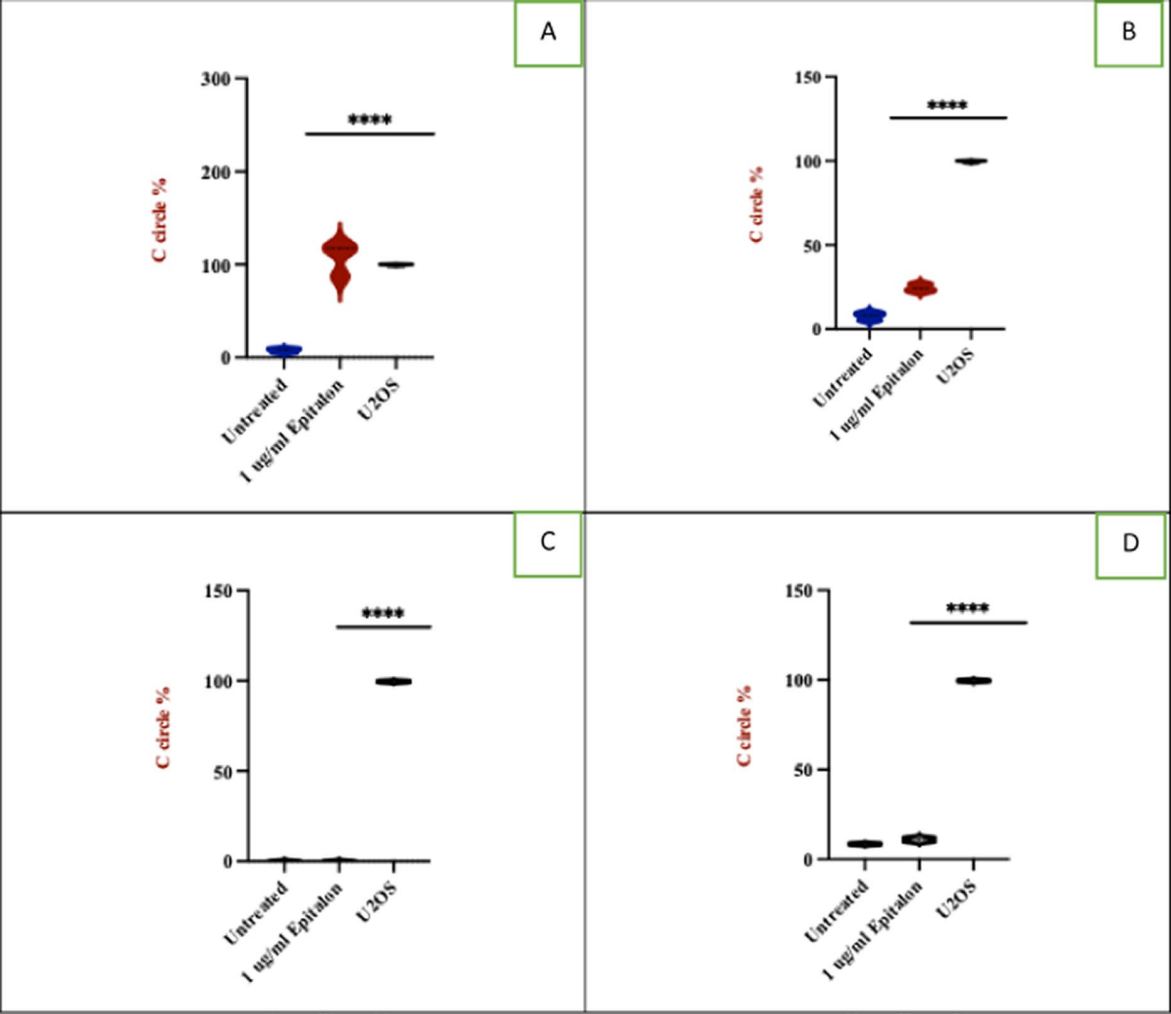
ALT activity in breast cancer cells and normal cells treated with epitalon. **A** and **B** ALT activity in 21NT and BT474 treated with 1 μg/ml of epitalon for 4 days. Untreated cells and the ALT positive U2OS were included as controls. **C** IBR.3 and **D** HMEC treated with 1 μg/ml of epitalon for three weeks. **E** Immunofluorescence to detect PML bodies in 21NT and BT474 cells treated with epitalon for 4 days. The colocalization PML bodies (green staining) within the nucleus (blue staining) for 21NT and BT474 treated with epitalon indicated the presence of ALT. PML bodies were detected using Lecia microscope with X 100 objective

The original article has been corrected.

