# Supplementary material for: Epitalon increases telomere length in human cell lines through telomerase upregulation or ALT activity
Source: Biogerontology. 2025 Sep 4;26(5):178. doi: 10.1007/s10522-025-10315-x (PMC12411320; doi:10.1007/s10522-025-10315-x)
Supplement: Supplementary file 1 — Supplementary file1 (PDF 387 KB) [file 10522_2025_10315_MOESM1_ESM.pdf]

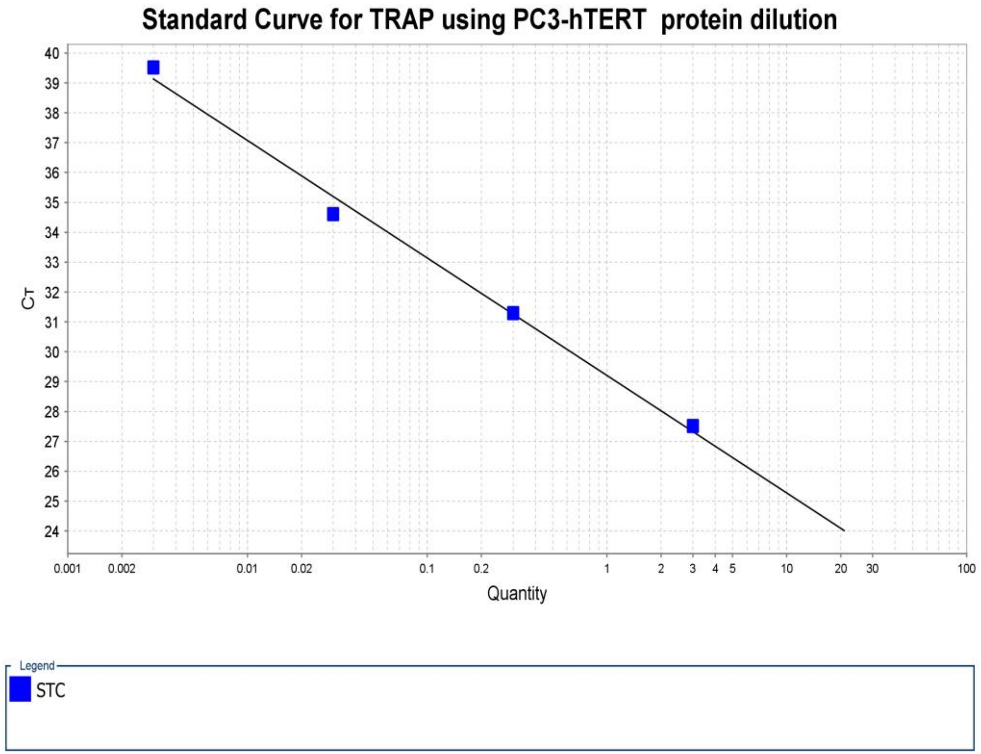
Standard Curve for TRAP using PC3-hTERT protein dilution
CT
40
39
38
37
36
35
34
33
32
31
30
29
28
27
26
25
24
0.001 0.002 0.01 0.02 0.1 0.2 1 2 3 4 5 10 20 30 100
Quantity
Legend
STC

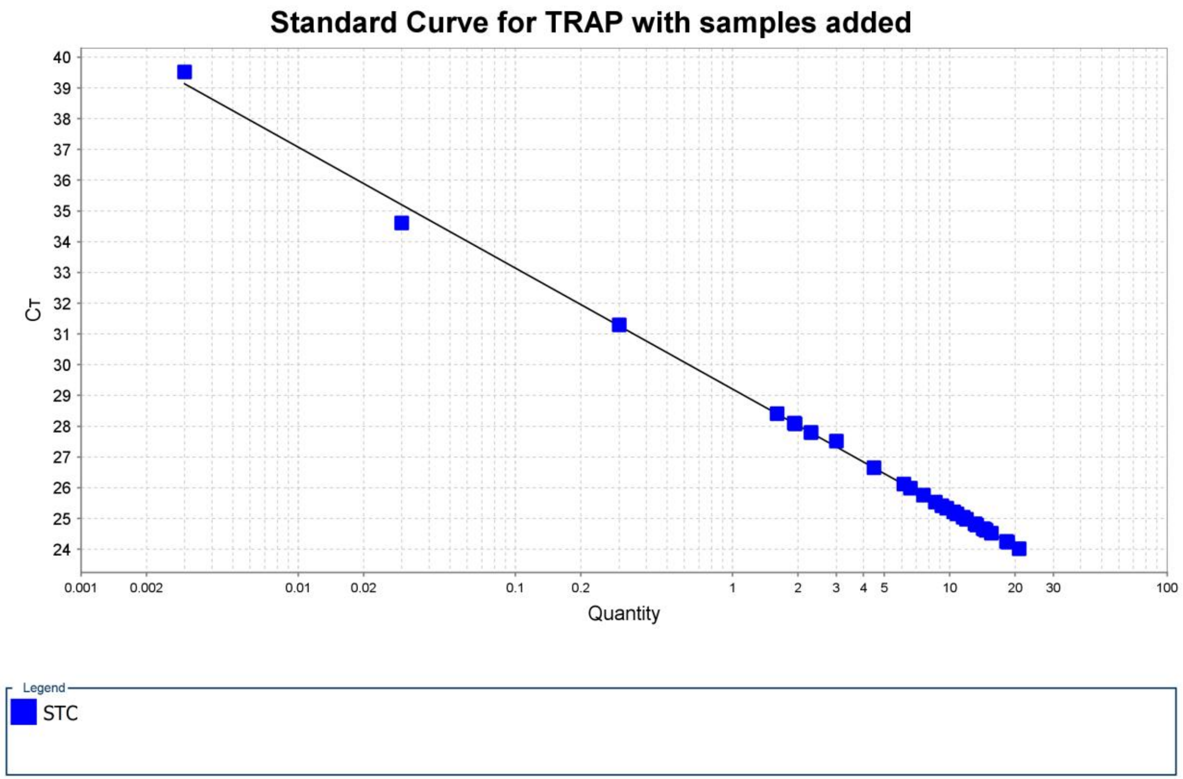
Standard Curve for TRAP with samples added
CT
40
39
38
37
36
35
34
33
32
31
30
29
28
27
26
25
24
0.001 0.002 0.01 0.02 0.1 0.2 1 2 3 4 5 10 20 30 100
Quantity
Legend
STC

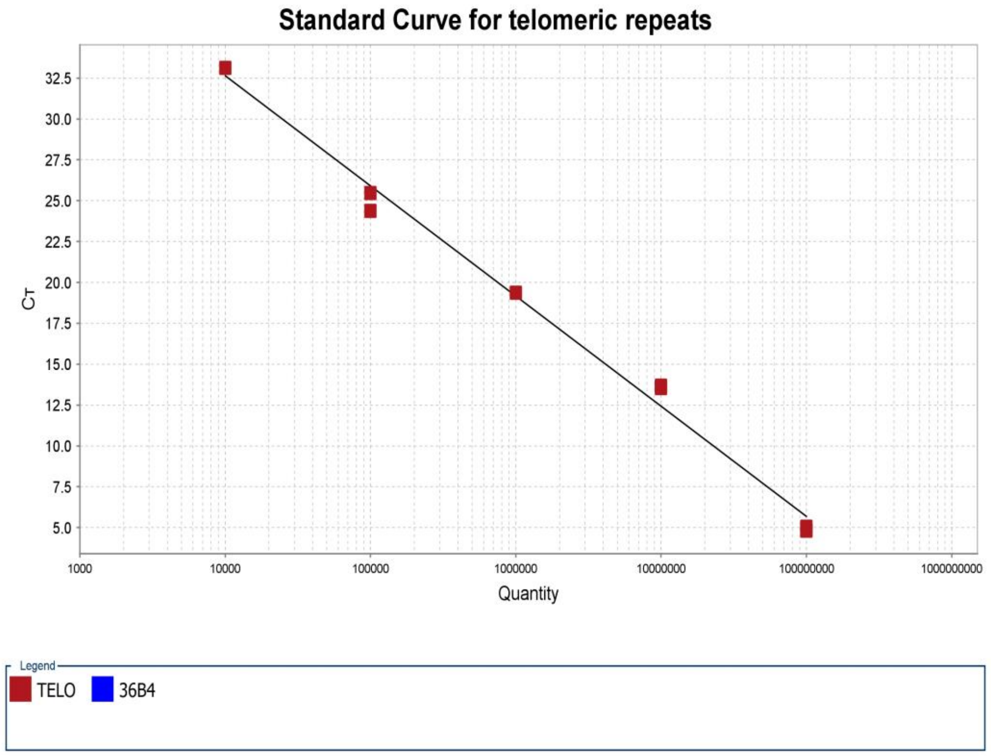
Standard Curve for telomeric repeats
Cт
Quantity
32.5
30.0
27.5
25.0
22.5
20.0
17.5
15.0
12.5
10.0
7.5
5.0
1000
10000
100000
1000000
10000000
100000000
1000000000
Legend
TELO
36B4

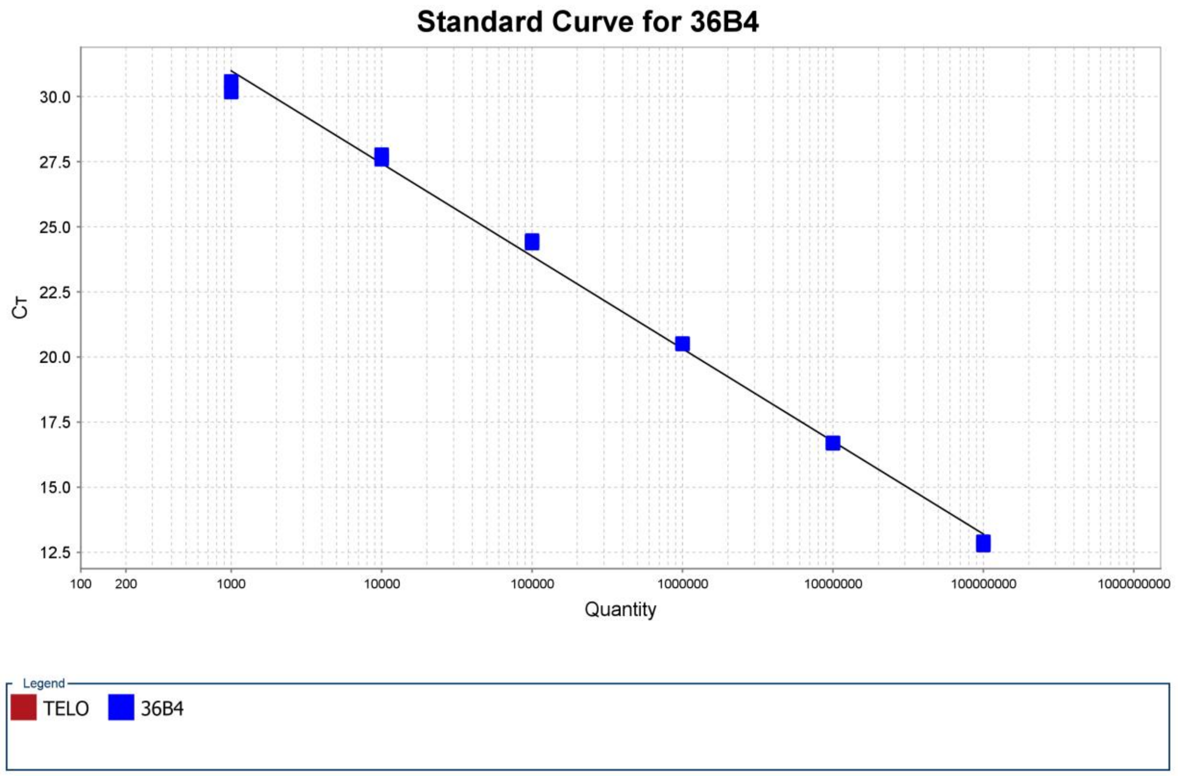
Standard Curve for 36B4
Cт
Quantity
30.0
27.5
25.0
22.5
20.0
17.5
15.0
12.5
100
200
1000
10000
100000
1000000
10000000
100000000
1000000000
Legend
TELO
36B4
